# Male sleeping aggregation of multiple Eucerini bee genera (Hymenoptera: Apidae) in Chapada Diamantina, Bahia, Brazil

**DOI:** 10.3897/BDJ.2.e1556

**Published:** 2014-09-12

**Authors:** Thiago Mahlmann, Juliana Hipólito, Favízia F. de Oliveira

**Affiliations:** † Instituto Nacional de Pesquisas da Amazônia, Manaus, Brazil; ‡ Universidade Federal da Bahia, Salvador, Brazil

**Keywords:** Long-horned bees, *
Melissodes
*, *
Melissoptila
*

## Abstract

Males of some groups of bees have to find a place outside the nests to sleep, sometimes forming “male sleeping aggregations”. Here we report the first record of “dense” male sleeping aggregation of two different genera of Eucerini bees observed in Bahia, Brazil. We discuss the possible aim of this kind of aggregation as well the plant utilized on aggregate.

## Introduction

While females of most solitary bee species spend the night inside their nests in construction, males have to find a place outside the nests to sleep, sometimes forming “male sleeping aggregations” (Evans and Linsley 1960, Oliveira and Castro 2002, Alves-dos-Santos et al. 2009). As far we know, first registered observations for Hymenoptera were made in the middle of XIX century, in which two species of Scoliid wasps were recorded (Westwood 1840) followed by the same behavior in one species in Apidae family (Cresson 1865).

 These aggregations may contain hundreds or even thousands of male individuals sharing the same sleeping site, where sometimes, but not as a rule, females also can be found together (Alcock 1998, Starr and Vélez 2009). According to Evans and Linsley 1960, aggregations can be divided into “dense (ball-like)” or “loose”, differing in the fact that there is no physical contact between insects in the latter one.

Dense aggregations usually are formed by a single species; however, few records with multiple species and genera can be found (e.g. Banks 1902, Rau and Rau 1916, Evans and Linsley 1960, Starr and Vélez 2009). Evans and Linsley 1960  found a loose aggregation with 21 species of wasps belonging to the families Crabronidae, Scoliidae, Sphecidae and Vespidae, as well as and 15 species of bees of the families Apidae, Megachilidae and Halicitidae). These authors noted that each taxon tended to be grouped separately in some specific place of the clump. Differences and patterns combine to the reasons for this behavior still remain as evidence gaps, requiring as much information as we can get to provide concrete assumptions.

In this paper we report the first occurrence record of a “dense” male sleeping aggregation including two different genera of Eucerini bees, *Melissodes* Latreille, 1829 and *Melissoptila* Holmberg, 1884.

## Materials and methods

The aggregation was observed in Ventura region, in Morro do Chapéu city, Chapada Diamantina, Bahia, Brasil (11°40′10.4″S; 40°58′12″W) in a fragment of deciduous or semi-deciduous forest (Veloso et al. 1991) on January 29, 2011 at 15h30. Agglomerate occurred in a dried inflorescence of a shrub (*Hyptis* sp., Lamiaceae), approximately 1 meter high from the ground.

All specimens observed were collected and deposited at Invertebrate Collection at Instituto Nacional de Pesquisas da Amazônia (INPA). The specimens were labeled with the following information: “BRA, Bahia, Morro do Chapéu, Ventura, 29.i.2011; 15h30; 11°40′10.4″S; 40°58′12″W, T.Mahlmann & J.Hipólito *Leg.*”; “03 males sleeping on dried flower”: Lamiaceae, aff. *Hyptis* sp.”

## Taxon treatments

### Melissodes (Ecplectica) nigroaenea

(Smith, 1854)

#### Materials

- **Type status:** Other material. **Occurrence:** catalogNumber: INPA s/n; recordNumber: s/n; recordedBy: T.Mahlmann et al.; individualCount: 2; sex: male; lifeStage: adult; behavior: 03 males sleeping on dried flower; establishmentMeans: native; preparations: pinned; disposition: good; **Taxon:** taxonID: Native; scientificName: Melissodes nigroaenea; acceptedNameUsage: Melissodes (Ecplectica) nigroaenea (Smith, 1854); kingdom: Animalia; phylum: Arthropoda; class: Insecta; order: Hymenoptera; family: Apidae; genus: Melissodes; subgenus: Ecplectica; specificEpithet: nigroaenea; taxonRank: species; scientificNameAuthorship: (Smith, 1854); **Location:** continent: South America; country: Brazil; countryCode: BRA; stateProvince: Bahia; municipality: Ventura; locality: Chapada Diamantina, Morro do Chapéu; locationRemarks: label transliteration: "BRA, Bahia, Morro do Chapéu, Ventura, 29.i.2011; 15h30; 11°40′10.4″S; 40°58′12″W, T.Mahlmann & J.Hipólito Leg.”; “03 males sleeping on dried flower”: Lamiaceae, aff. Hyptis sp."; verbatimCoordinates: 11°40′10.4″S 40°58′12″W; decimalLatitude: -11.669556; decimalLongitude: -40.970000; georeferenceProtocol: GPS; **Identification:** identifiedBy: T.Mahlmann; dateIdentified: 2014; **Event:** samplingProtocol: sweeping; eventDate: 2011.i.29; eventTime: 15h30; year: 2011; month: 1; day: 29; **Record Level:** language: pt; collectionCode: Insects; basisOfRecord: PreservedSpecimen

### Melissoptila aff. bonaerensis

Holmberg, 1903

#### Materials

- **Type status:** Other material. **Occurrence:** catalogNumber: INPA s/n; recordNumber: s/n; recordedBy: T.Mahlmann et al.; individualCount: 1; sex: male; lifeStage: adult; behavior: 03 males sleeping on dried flower; establishmentMeans: native; preparations: pinned; disposition: good; **Taxon:** taxonID: Native; scientificName: Melissoptila aff. bonaerensis; acceptedNameUsage: Melissoptila aff. bonaerensis Holmberg, 1903; kingdom: Animalia; phylum: Arthropoda; class: Insecta; order: Hymenoptera; family: Apidae; genus: Melissoptila; specificEpithet: aff. bonaerensis; taxonRank: species; scientificNameAuthorship: Holmberg, 1903; **Location:** continent: South America; country: Brazil; countryCode: BRA; stateProvince: Bahia; municipality: Ventura; locality: Chapada Diamantina, Morro do Chapéu; locationRemarks: label transliteration: "BRA, Bahia, Morro do Chapéu, Ventura, 29.i.2011; 15h30; 11°40′10.4″S; 40°58′12″W, T.Mahlmann & J.Hipólito Leg.”; “03 males sleeping on dried flower”: Lamiaceae, aff. Hyptis sp."; verbatimCoordinates: 11°40′10.4″S 40°58′12″W; decimalLatitude: -11.669556; decimalLongitude: -40.970000; georeferenceProtocol: GPS; **Identification:** identifiedBy: T.Mahlmann; dateIdentified: 2014; **Event:** samplingProtocol: sweeping; eventDate: 2011.i.29; eventTime: 15h30; year: 2011; month: 1; day: 29; **Record Level:** language: pt; collectionCode: Insects; basisOfRecord: PreservedSpecimen

## Discussion

Three male specimens belonging to distinct species and genera of Eucerini were observed and collected. The aggregation was formed by two males of Melissodes (Ecplectica) nigroaenea (Smith, 1854) (Fig. 1a) and one male of Melissoptila aff. bonaerensis Holmberg, 1903 (Fig. 1b). The specimens formed a small “dense aggregation” by holding the down side of the drayed capitulum of the shrub and remain suspended by their mandibles maintaining their legs close to the body (Fig. 2a).

Formation was not observed until it was totally dark, making impossible to know the entire aggregation processes. It is possible, however, that more bees had joined the formation after our observations.

Instead what has been reported by stingless bees in males “congregations” without much definite information (Santos et al. 2014), several hypotheses have been formulated as possible explanation for this kind of behavior in solitary bees. Among the parsimonious hypothesis is the dilution effect as suggested by Alcock 1998,  by observing a species of the genus *Idiomelissodes* LaBerge (Apidae, Eucerini). This hypothesis implies that, as long as individuals are equally spaced and at the same distance from the predator, all of them have an equal probability of being targeted and killed during an attack (Vine 1971). This can be advantageous for all species involved, specially in conditions were individuals are resting and not able to easily escape.

Males reported here have long antennae and brownish hairs with an integument predominantly dark brown, which compared to the hairy aspect and pale color of the inflorescence where the aggregation was formed could indicate an nice strategy of camouflage  (Fig. 2b).

Despite there are several records on different types of sleeping aggregations on literature, new records as the one reported here may help to better understand of reasons of this behavior.

## Supplementary Material

XML Treatment for Melissodes (Ecplectica) nigroaenea

XML Treatment for Melissoptila aff. bonaerensis

## Figures and Tables

**Figure 1a.**
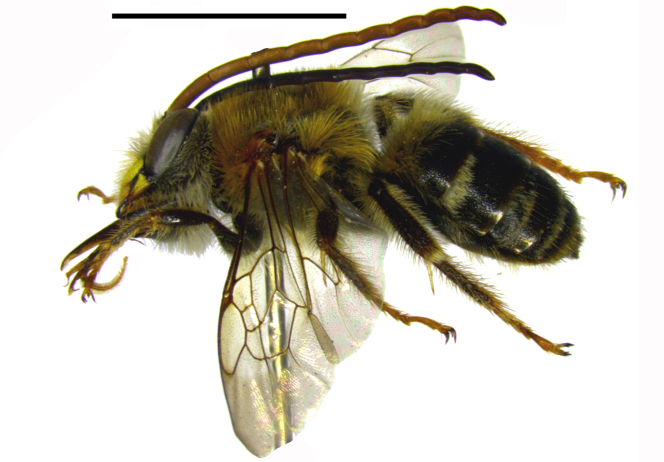
Melissodes (Ecplectica) nigroaenea

**Figure 1b.**
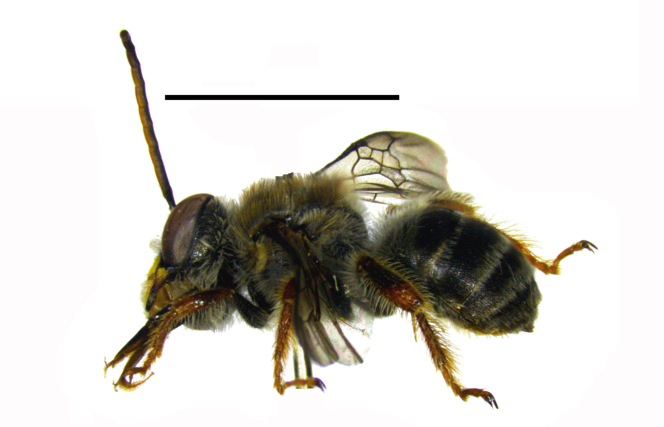
Melissoptila aff. bonaerensis

**Figure 2a.**
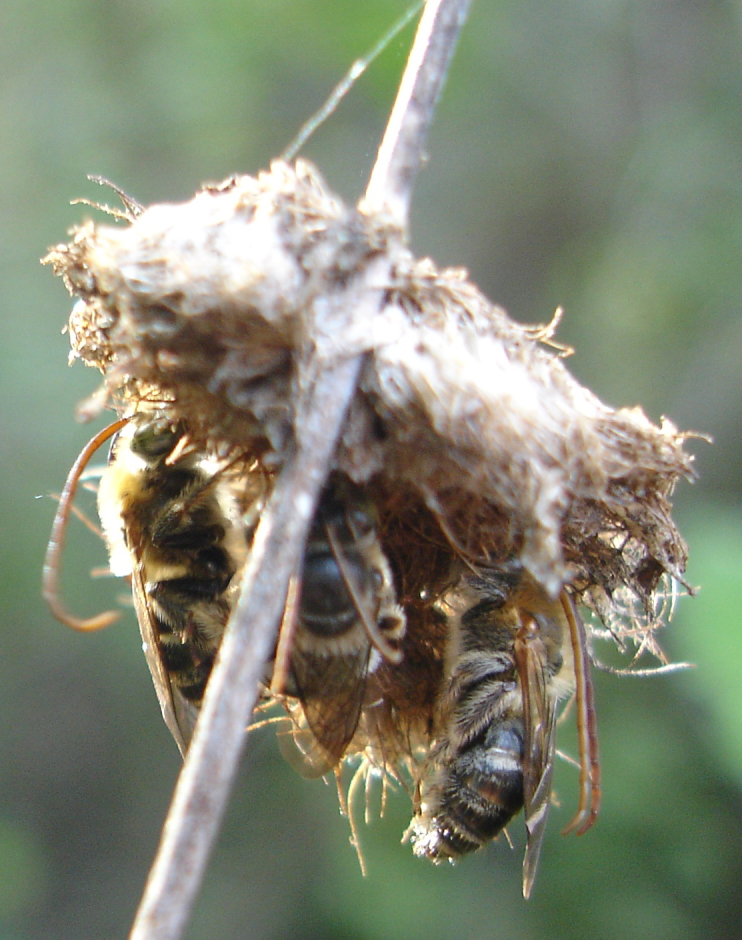
specimens holding the down side of the capitulum by their mandibles

**Figure 2b.**
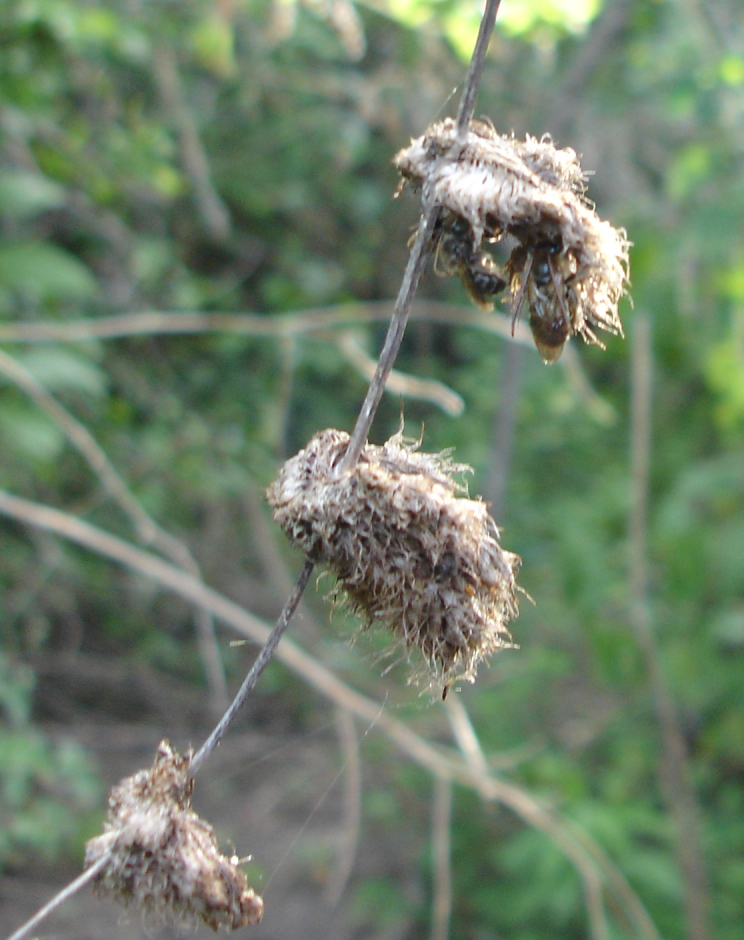
specimens hidden into the dried inflorescence
